# Reproductive autonomy of women living with multiple myeloma participating in a pregnancy prevention program

**DOI:** 10.1186/s12978-025-02182-z

**Published:** 2025-11-20

**Authors:** J. M. Wigle, M. Ramasamy, A. McCurdy, L. V. Dias, H. Mian, I. Sandhu, C. Pritlove

**Affiliations:** 1 Applied Health Research Centre, Li Ka Shing Knowledge Institute, Unity Health Toronto, Toronto, Canada; 2 Ottawa Hospital Research Institute, Ottawa, ON Canada; 3 Department of Oncology, McMaster University, Hamilton, ON Canada; 4 Department of Oncology, University of Alberta, Cross Cancer Institute, Edmonton, Canada; 5 Dalla Lana School of Public Health, Social and Behavioural Health Sciences, University of Toronto, Toronto, Canada

**Keywords:** Reproductive autonomy, Multiple myeloma, Females of childbearing potential, Reproductive justice, Reproductive rights, Controlled distribution programs, Pregnancy prevention programs

## Abstract

**Background:**

Multiple myeloma is an incurable hematologic cancer that primarily affects older adults. Females of childbearing potential represent a small but uniquely affected proportion of the multiple myeloma population. The immunomodulatory agents (thalidomide, lenalidomide and pomalidomide) are highly effective treatments in improving prolonged periods of deep remission and long-term survival in Multiple Myeloma, is dispensed through controlled distribution programs that require pregnancy monitoring for females of childbearing potential to reduce the risk of fetal exposure. There is limited understanding of the impact of pregnancy prevention and monitoring measures on the reproductive health, autonomy, and rights of women living with multiple myeloma.

**Methods:**

This critical qualitative study is informed by a descriptive methodological approach, and a feminist reproductive justice theoretical framework informed the data analysis and interpretation. We employed purposive sampling to identify and conduct interviews with females of childbearing potential, living with multiple myeloma, that have experience participating in a controlled distribution program.

**Results:**

This study reflects the experiences of 15 females of childbearing potential living with MM, all of whom identified as women and ranged in age from 33–50 at diagnosis. Participants were situated in provinces across Canada, with most identifying as White, married, and of higher socioeconomic status. Findings illuminate a multitude of ways in which controlled distribution programs imposed threats to the reproductive health and autonomy of these women. Although many participants acknowledged the importance of preventing fetal exposure to teratogenic medications, the prescriptive and controlling nature of hyper-vigilant pregnancy monitoring programs and practices imposed significant burden and constraints on females of childbearing potential. Key analytic themes highlight the perceived paternalistic nature of controlled distribution programs, the systemic distrust of females of childbearing potential, and women’s actions and advocacy efforts to (re)claim their reproductive agency.

**Conclusion:**

Participant-informed adaptations to the design and delivery of pregnancy monitoring and prevention requirements in existing controlled distribution programs to promote the reproductive autonomy and agency of females of childbearing potential are both necessary and feasible. Key recommendations include increased provision of timely, comprehensive information and education, psychosocial support, as well as modifications to programs and regulatory bodies to recognize women as trustworthy and capable of autonomous, reproductive health decision-making.

**Supplementary Information:**

The online version contains supplementary material available at 10.1186/s12978-025-02182-z.

## Abstract (plain language summary)

Multiple myeloma is an incurable type of blood cancer that primarily affects older adults. Females of childbearing potential represent a small but uniquely affected proportion of the multiple myeloma population. Recent advances in treatment have contributed to long-term improvements in survival yet pose potential risks for fetal exposure among females of childbearing potential. Treatments for multiple myeloma, particularly thalidomide and its derivatives lenalidomide and pomalidomide (otherwise known as immunomodulatory agents (IMiDs)), have led to the establishment of controlled drug distribution programs to prevent fetal exposure, requiring pregnancy monitoring for females of childbearing potential. However, there is limited understanding of the impact of pregnancy prevention and monitoring measures on the reproductive health, autonomy, and rights of women living with multiple myeloma. In this descriptive qualitative study, informed by feminist reproductive justice theory, we conducted interviews with 15 females of childbearing potential living with multiple myeloma that have experience participating in a controlled distribution program. Our findings demonstrate that although participants acknowledged the importance of preventing fetal exposure to IMiDs, controlled distribution programs represent a significant burden and threat to individuals’ reproductive health and autonomy. Themes produced and explored in this paper highlight the perceived paternalistic nature of controlled distribution programs, distrust of females of childbearing potential, and women’s actions and advocacy towards reclaiming their reproductive agency. Participant-informed adaptations to the design and delivery of pregnancy monitoring and prevention requirements in existing controlled distribution programs are provided.

**Supplementary Information:**

The online version contains supplementary material available at 10.1186/s12978-025-02182-z.

## Background

Multiple myeloma (MM) is a hematologic cancer that primarily affects older adults, with a mean age of 70 years at the time of diagnosis [56]. MM is the 21 st most common type of cancer globally [26], and 16th in Canada; it accounts for 1.9% and 1.4% of new cancers among males and females, respectively [12]. An estimated 10% of cases occur in individuals under 50 years of age, yet younger patients remain underrepresented in the literature [53].

Recent improvements in survival for individuals living with hematologic cancers are attributed to earlier diagnosis, introduction of novel therapies, optimizing existing treatments, and improvements in supportive care [23]. Evolutions in MM treatment, particularly proteasome inhibitors, immunomodulatory agents (IMiDs), monoclonal antibodies, and high-dose therapy with auto-transplantation have dramatically improved long-term survival, achieving prolonged periods of deep remission [36]. Thalidomide and its derivatives, lenalidomide and pomalidomide, are IMiD therapies widely used in MM treatment, which have contributed substantially to this progress [14]. However, to minimize the risk of teratogenic effects and prevent fetal exposure to IMIDs, patient access to these drugs is regulated via controlled distribution programs (CDP) (e.g., *RevAid* or *Aposecure in Canada,* and *Risk Evaluation and Mitigation Strategies (REMS)* in the United States). These programs have specific requirements for FCBP, such as routine pregnancy monitoring and use of two forms of contraception^1^ (see Supplementary File 1 for examples of CDPs and eligibility requirements) [2, 3, 40, 57]. Data from the *Revlimid* patient registry in the United States in 2005–2015, estimates that 7% of all females and 3% of all patients receiving IMiDs were categorized as FCBP (with a median age of 47 years) [13]. Given innovations in treatments and the associated emergence of regulatory efforts within the MM landscape [34, 36], understanding the unique experiences of accessing treatments amongst females of childbearing potential (FCBP)^2^ is warranted.

Thalidomide was initially marketed in the late 1950s and early 1960s as a sedative-hypnotic agent widely used to treat nausea in pregnant individuals until 1961, when it was removed from the market after confirmation it caused miscarriages, stillbirths, and severe congenital malformations (phocomelia), affecting thousands of infants [28, 34]. As a result, systematic changes to pharmaceutical development and toxicity testing were introduced in the 1960 s [28]; and the continued approval and use of known teratogens has contributed to the establishment of strict risk minimization strategies and regulations, including CDPs [2, 3, 34, 40, 57]. These programs supply medications when specific predefined criteria are met and involve oversight of the distribution chain and educational materials for enrolled patients, identification and registration of all institutions (e.g., pharmacy, hospital, distributor), and training of health care professionals (e.g., registered clinicians/prescribers and pharmacists) [34]. Several controlled distribution programs require mandatory pregnancy prevention and monitoring to regulate teratogenic medications and to prevent the risk of congenital abnormalities and malformations during pregnancy, including phthalimides (e.g., thalidomide, lenalidomide and pomalidomide) [34], isotretinoin, and acitretin [50].

Despite limited evidence of the effectiveness of CDPs on the rates of pregnancy, abortion, or fetal exposure among FCBP [15, 24, 49, 54], substantial ethical, logistical, and health system concerns have been identified in the literature [38, 48, 54]. Evidence from other regulatory and risk minimization efforts, such as *iPLEDGE*, a CDP for isotretinoin, highlight potential restrictions on participating individuals’ autonomy (and reproductive autonomy), and that CDPs contribute to disproportionate delays, burden, and interruptions based on individuals’ gender, race, and socioeconomic status [38, 48]. The lived experiences of MM patients and extensive work involved in managing their treatment, side effects, and quality of life has already been explored in literature, using qualitative methods [37, 39]. There is a dearth of evidence on the diverse physical and psychosocial implications of participating in a CDP and its influence on the quality of life and overall wellbeing among FCBP. Further, the specific impact of participation in a CDP on the reproductive agency, autonomy, and rights of FCBP living with MM remains unexplored. As a result, this paper seeks to address this gap in knowledge using a critical qualitative approach, informed by a reproductive justice, to examine the impact of these controlled distribution programs on the sexual and reproductive health and rights of women living with MM.

## Methods

### Theoretical framework

Theory may be integrated at any point in the research process [44]. Although our initial descriptive study design and data generation were not informed by a specific theoretical orientation, we chose to integrate theory as an “organizational framework for both the interpretation and re-presentation of data” after our initial analysis ([44], p. 216). After engaging in open, inductive, and reflexive thematic analysis [9] multiple codes, including: “[CDP] requirements”, “invisibility of women’s reproductive autonomy”, and the “[CDP] and the burden of proof” underscored the complexity of women’s reproductive agency, autonomy, and rights within the context of participating in a CDP while living with MM. Therefore, we integrated a critical feminist, reproductive justice theoretical lens as a framework to guide and inform further analysis and interpretation of study data.

Reproductive justice theory emphasizes that women, Two Spirit, trans, and non-binary people’s reproductive autonomy and decisions on whether to have children (or not) and use of sexual and reproductive health services and contraception are shaped by underlying social, economic, and political contexts [33, 42]. Grounded in Black feminist scholarship on women’s reproductive rights [42, 43], this theoretical approach allows researchers to examine how individuals’ reproductive health are shaped by “socio-political complexities and criss-crossing power dynamics that proceed along multiple interconnected axes of difference (e.g., classism, sexism, racism, heterosexism)…and provides insight into systems of difference and inequality*”* ([33], p. 4). It also helped our consideration of individuals’ historical, legal, and technological lived reproductive realities and how they seize spaces and opportunities to effectively and autonomously manage their fertility [43]. It connects individual reproductive injustices to systemic sources of power and oppression, by challenging assumptions and the dominant rhetoric of norms/beliefs related to fertility and childbearing amongst individuals living with a terminal condition. This approach has been applied previously by Sundstrom et al. [52] to explore women’s experiences with the human papillomavirus, and cervical cancer prevention. It has also been employed and offers insights on how individuals’ race and insurance status undermine their timely access to vital SRH services, including fertility counselling prior to cancer treatments [31]. A central tenet of this theory is its social justice and praxis-orientation to expose and challenge laws, policies, and practices that perpetuate racial, gender, and class-based inequities that restrict individuals’ bodily autonomy, and their reproductive decision-making to catalyze social change [33, 43]. From this perspective, we move beyond framing individuals as passive recipients of and participants in a controlled distribution program, to recognizing their everyday acts of resistance and advocacy for systemic change.

### Study design

A critical qualitative approach was employed in this research to explore the lived experiences of FCBP living with MM, and the impact of participating in a CDP on their quality of life. In particular, we employed a qualitative descriptive study design, which recognizes the subjective nature of research and grounds our findings in women’s diverse experiences living with MM. Qualitative descriptive approaches are frequently employed in healthcare research and offer important tools and insights to examine areas where limited research is available, explore participants’ lived experiences and to address pressing clinical, service, or policy challenges in practice [19]. Using this approach, we generated data to explore the “who, what, and where” [46] of women’s individual and subjective experiences of participating in a CDP for MM.

In addition, we acknowledge the centrality and “creative presence” of the researcher in shaping qualitative inquiry ([20], p. 4). Our worldviews, research interests, and decisions made throughout this study are informed by our individual and collective epistemological and social positions/identities, as an interdisciplinary team of educated and predominantly female health researchers and clinicians. Moreover, as critical feminist scholars (JW/CP/MR/LD) and MM clinical investigators committed to equity in MM care (AM, HM, IS), we seek to examine underlying social factors and systems of power that affect the reproductive choices and health of women living with MM.

### Sampling and recruitment

We used purposive sampling strategies, including maximum variation sampling [45], to identify individuals living with MM and experience with IMiD therapies. We aimed to capture diversity among participants’ experiences by socioeconomic status, race, ethnicity, geographic location, sexuality, and other aspects of social identity. We also employed snowball sampling [22], inviting participants to share information about the study with other individuals in their networks.

Multiple recruitment strategies were employed including collaborating with Myeloma Canada (a national organization focused on patient advocacy and support) (Myeloma [35]) and the Canadian Myeloma Research Group (CMRG) (a national research and clinical trials organization) [17] to share study information with their membership via email, monthly newsletters, social media, as well as disseminating study information with relevant Myeloma Canada support groups. Prior to screening, participants were emailed a study information and consent letter outlining the research objectives, inclusion criteria, as well as the potential risks and benefits of their involvement. We screened potential participants for eligibility according to the inclusion criteria (self-identify as living with MM, experience with IMiD therapies in the last five years, considered to be a FCBP according to CDP program standards, over 18 years of age, and speak English) and scheduled interviews. All participants who contacted the research coordinator, and who were deemed eligible, consented to participate and completed a study interview. Ethics approval for this study was provided by the St. Michael’s Hospital Research Ethics Board (REB #21-251C). All participants provided verbal informed consent.

### Data generation

Semi-structured interviews were conducted to explore the experiences of FCBP living with MM, and their participation in a CDP. Interview guides examined the impact of their diagnosis on their lived realities and their social support networks, their experiences with treatments and engagement in treatment-related decisions, as well as their specific experiences participating in a CDP. Interview guide development was informed by a review of literature and research objectives. Four open-ended questions examined: i) the impact of a MM diagnosis on individuals’ lived realities and social support networks; ii) lived experiences with treatments and engagement in treatment-related decisions; iii) specific experiences participating in a CDP; and iv) identifying unmet care needs and recommendations. Additional probes or prompts for further details, description, or clarification were also employed. Two researchers (JW and CP), with extensive doctoral-level qualitative methodological training and expertise, conducted a total of fifteen one-to-one interviews with FCBP between December 2021 and October 2022 via telephone or Zoom and all participants completed a demographic questionnaire at the conclusion of the interview. JW and CP met after conducting the initial 1–2 interviews to collaboratively discuss and finalize the interview guide and met regularly while generating data to discuss initial interpretations. Interviewers did not have existing relationships with any study participants prior to their engagement in this research.

### Data analysis

All interviews were audio recorded using digital (encrypted) audio recorders, transcribed verbatim by a professional transcriptionist and quality checked by the research team for accuracy. During the quality assurance process, any personal or identifying information was removed by the study team. Interviews ranged in length from 62 to 281 min, lasting on average 92 min. Reflexive thematic analysis was conducted to identify, analyze, and report on patterns and themes in the data. Data was initially analyzed using an inductive approach, as we reviewed and familiarized ourselves with the data, produced initial codes, critically examined collated data for themes, reviewed themes, and defined and named themes [8, 9]. Several team members (CP, JW & MR) independently coded qualitative transcripts and collaboratively produced a codebook to define and refine code names, definitions, quotes, and analytic notes. We revised the codebook in an iterative way, adjusting code names and definitions as interview data were added and as our thinking evolved through team-based discussions. We employed memo writing as a reflective “procedural and analytical” strategy to support “conceptual leaps from raw data” towards producing theme development and theorization ([7], p. 68). For instance, in generating and revising the codebook we used memoing to document our initial interpretations, the evolution of codes, and to draw connections across codes and the categorization of data. During analysis, we observed rich discussions embedded within codes related to participants’ reproductive decisions, contraceptive use, and impact of their participation in a CDP on their reproductive identity,^3^ agency, and autonomy. Therefore, we also employed abductive reasoning,^4^ to draw insights from reproductive justice theory, to inform our interpretation and theorization of participants’ agency, power, and control of their reproductive health and decisions. This analytic process allowed us to group relevant codes to generate higher order concepts of “generalized statements” synthesizing the lived experiences of women enrolled in a controlled distribution program for multiple myeloma ([6], p. 109). We used empirical data to inform, define, and expand our understanding of well-known concepts, such as “reproductive autonomy”, “agency” and “trust” in terms of the structural systems of power and oppression that shaped women’s experiences of participating in a CDP. Several measures to ensure quality and rigour of qualitative research were utilized, such as outlining the research rationale, rich description/rigour, resonance, and contribution to scholarship [55]. Moreover, the concept of “information power” was also considered give the specific eligibility criteria, adoption of a theoretical framework, rich interview dialogue, and analytic procedure ([30], p. 1753).

We employed NVivo 12 computer software to manage data, facilitate qualitative coding, and to produce summary code documents. In addition, we further reviewed transcripts to examine how other axes of social identity, including race and income, may have shaped participants’ lived experiences of reproductive agency and autonomy due to their participation in a CDP.

### Ethical considerations

This study was reviewed and approved by the Research Ethics Board at Unity Health Toronto (REB #21-251C). Verbal informed consent was obtained from all study participants, and the research coordinator (LD) recorded consent prior to conducting each interview. All data was stored on an encrypted hospital server, password protected, and accessed only by the research team.

## Results

### Participant characteristics

This research reflects a range of sociodemographic positions amongst FCBP living with MM. All participants identified as women and female (100%), ranging in age from 33–50 years at the time of their MM diagnosis. Ten participants (67%) identified as White (European), and most were married (73%), with child(ren) at home (80%). Although half of the study participants lived in Ontario, there was also representation across four provinces/territories, and the United Kingdom. Overall, most participants lived in either medium (20%) or large (53%) urban centres. Further, most participants identified as having a high family income (80%)^5^ and post-secondary education (80%). Detailed demographic information for the study participants is summarized in Table 1.

**Table 1:** Demographic characteristics^1^

Characteristics	Frequency of participants (*n*)	Percentage of participants (%)
Age at time of diagnosis (years)		
< 39	7	46.7
40–59	8	53.3
Sex		
Female	15	100
Gender		
Woman	15	100
Ethnic background		
White	11	73.3
Indigenous and racialized	4	26.7
Living situation		
Married	11	73.3
Unmarried (e.g., single or divorced)	4	6.7
Have children at home	12	80.0
Place of residence^2^		
Small population centre	4	26.7
Medium population centre	3	20.0
Large urban population centre	8	53.3
Total before-tax family income in 2020		
< $29,000	3	20.0
> $70, 000	12	80.0
Education		
Completed some/all high school	3	20.0
College diploma	2	13.3
Undergraduate degree	3	20.0
Graduate degree or greater	7	46.7
Current employment status		
Employed (full or part-time)	5	33.3
Sick leave	8	53.3
Other	2	13.3

^1^To ensure a cell size of ≥ 2 due to small sample size, we have collapsed some categories from the original survey options to maintain confidentiality

^2^Place of residence is classified by population size (Rural area: population < 1,000, small population centre: population 1,000–29,999, medium population centre: population 30,000- 99,999, large urban population centre: population > 100,000)

### Themes

**Fig. 1:**
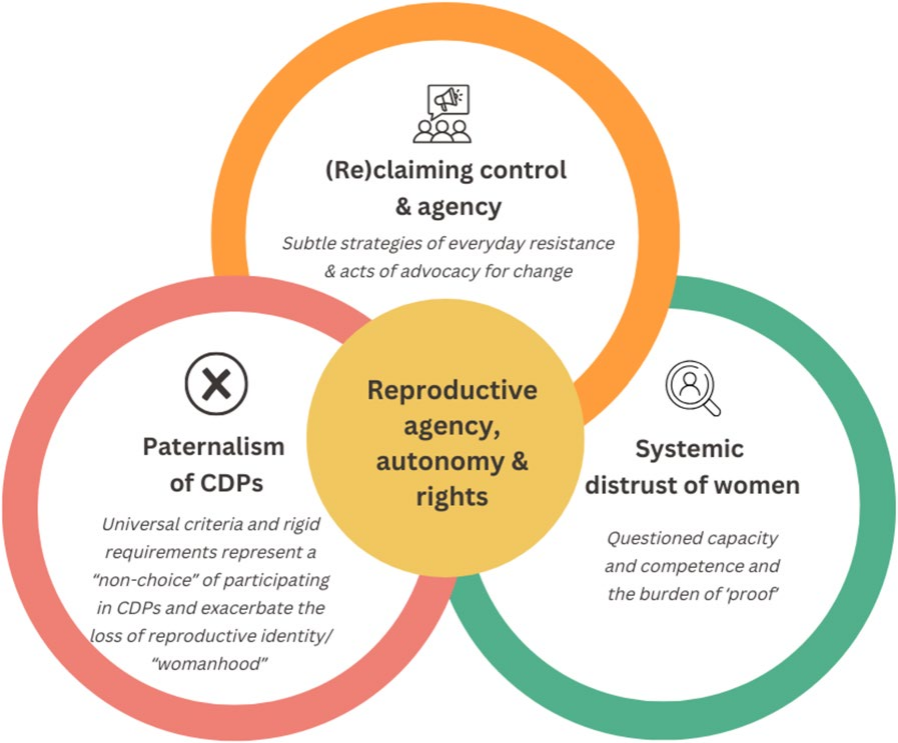
Analytic themes produced illustrating the reproductive agency, autonomy, and rights of females of childbearing potential with multiple myeloma participating in a controlled distribution program

Women living with MM, who were considered FCBP, experienced unique challenges related to their reproductive health and autonomy due to their enrolment in a CDP. Accounts of their experiences within CDPs illuminated the controlling nature of what they believed to be hyper-vigilant monitoring and risk mitigation practices. Guided by a reproductive justice lens, key analytic themes produced and discussed in turn represent underlying forces that shape the reproductive agency, autonomy, and rights of women living with MM, including: i) paternalism of CDPs; ii) systemic distrust of women; and iii) (re)claiming control and agency (see Fig. 1).

### Paternalism of CDPs: *“This program is policing a woman’s body”*

For most FCBP in this study, CDPs represented a “paternalistic” approach to minimizing teratogenic effects, while circumscribing women’s reproductive agency and autonomy. Universally applied and non-flexible program requirements (e.g., pregnancy monitoring and contraceptive use) for all FCBP, paired with a lack of clear and transparent communication about these requirements when making treatment-related decisions, reinforced feelings of stripped autonomy. These feelings of lost autonomy were exacerbated by perceptions that adherence with strict requirements and vigilant monitoring was a *“non-choice”*, trading their reproductive autonomy for life-prolonging medications. Accompanied by physical changes imposed by a MM diagnosis and accompanying treatments, this sense of stripped reproductive autonomy further threatened participants’ (reproductive) identity.

#### Universal criteria and rigid requirements

Nearly all participants reported experiencing early-onset, treatment-induced menopause, yet this did not exclude them from the pregnancy monitoring/prevention requirements of the CDP. Against this backdrop, participants described a lack of communication and general confusion on the purpose of the CDP, eligibility criteria for being categorized as a FCBP, duration of this classification, as well as prospects for challenging this status. As the participant below states:

*I guess I just thought, you, you know, as time goes on, the requirement will be eased, because I'm obviously in, you know, the longer I'm in ah menopause, ah then, then, you know, they'll ease the requirements. So, and they never did. So, no one, ah, yeah, I didn't, I didn’t know that [pregnancy monitoring] would keep going on and on and on* (Participant 15)

Some women described substantial efforts (with support from their health care providers) to ‘prove’ through diagnostic testing that they no longer qualified as a FCBP; however, these requests failed to result in changed status for the participants in this study. Participants explained that they received limited explanation from the CDP about the evaluation process and/or the grounds upon which their request for changed status was being denied. As a result, many had little understanding of how long they would be required to participate in the CDP and suggested that more clearly informing women at the beginning of their enrolment would be particularly beneficial to support informed decision making and set appropriate expectations. One participant described the extensive work to (unsuccessfully) petition their status as a FCBP, as well as the inadequacies of the current ‘one-size-fits-all’ approach of pregnancy monitoring programs:

*The biggest challenge I have with it right now is that it doesn't seem to matter what documentation either my oncologist sends in or my gynecologist sends in, none of that seems to matter, to say 'Hey, I am forty-six. I am officially in menopause. I am not, (laugh) I’m not at a pregnancy risk.' None of that seems to matter.* (Participant 01)

It was felt that the standardized approach to mandatory contraceptive use (two forms of birth-control) and pregnancy monitoring adopted within CDPs inadequately accounted for women’s unique experiences, relationship status, and/or reproductive decisions/preferences (to not have (more) children) made prior to diagnosis. This represented a source of resentment and frustration for many women in this study. Participants highlighted that the CDPs fail to recognize women’s reproductive health preferences and decisions as “valid”, standalone reasons for their exclusion from strict monitoring requirements:

*I personally am not interested in having children at this stage in my life. And that needs to be a valid answer in some cases. Like, it just, it does. That, that is my choice, and it, it, it doesn't matter. At this point, it doesn't seem to matter that my choice and my healthcare team backing me up on the physical side of things, doesn't matter, at all. So, it's, that's frustrating.* (Participant 01)

In addition, many participants described receiving limited information related to contraceptive options to meet program requirements. Some women expressed concern about hormonal contraception given their diagnosis of MM but felt that few healthcare providers or CDP representatives were not able to speak to such considerations, which made decision-making about contraception incredibly difficult. Some were not provided advance or sufficient notice about the CDP requirement to have two forms of contraception in place before initiating IMiD treatment, leaving them insufficient time to access certain options (e.g., vasectomy for partner or tubal ligation), and ultimately contributing to unnecessary delays in treatment onset:

*If I had known this like six months ago, my husband probably would have just got a vasectomy. Like and then I wouldn't have to deal with the um blood tests as much or like, anyways, so I already talked to him about it. Like, we wish we had known […] so I had to delay the lenalidomide, to ensure, like, the two forms were in place and all that. So, um yeah. I just wish they had told me ahead of time. Cause my husband probably would have just got on the waitlist and got that done before.* (Participant 09)

The lack of timely information on the eligibility criteria for FCBP and women’s enrolment in the CDP, inconsistent communication of (available) contraceptive options, and an overall failure to acknowledge participants’ heterogeneous lived reproductive realities and decisions (e.g., choosing not to have more/any children prior to diagnosis) demonstrate some of the ways that existing regulations within CDPs can undermined women’s personal reproductive autonomy and choices.

#### The *“non-choice”* of participating in CDPs

Recognizing the problematic history of Thalidomide and the importance to taking the necessary precautions, including pregnancy prevention, while on IMiD therapies, many participants critically questioned the appropriateness of CDPs’ mandating and monitoring contraception use, saying: *“government and private corporations don't belong in bed with my husband and I…”* (Participant 06). For some, the program design and approach were reminiscent of historical examples of policing and controlling women’s bodies and reproductive autonomy:

*I think the program is paternalistic. The program is constructed in a way that the drug company and Health Canada clearly don't trust […] this program is policing a woman's body. There have been, you know, repeated examples over time of women's bodies being policed: dress codes, abortion laws, and now this is another type, er, example of rules being put in place to police a woman's fertility…this program takes away a woman’s ability to manage her own reproductive system, control her fertility. It takes away a woman’s ability to autonomously manage her own reproductive rights* (Participant 06).

Ethical concerns were raised by some who saw the withholding of life-prolonging medication in instances of non-compliance with program requirements (e.g., missed pregnancy test) as being coercive. As one participant shared:

*The biggest frustration with [the controlled distribution program] is probably the, the strict rules about two forms of birth control and getting a pregnancy test every month, and, you know, having to review that, it's just, I don't, I don't really understand why it's necessary to that level. Like, I do agree that it is important to, just you know, be careful and monitor. I don't even have a problem doing the pregnancy test. But I just don't know if it needs to be in the form of like, withholding [medications] until it's done.* (Participant 11)

Several examples of limited engagement in directing treatment trajectories were outlined: *“I always felt stupid for asking questions”* and *“I always felt like it was um, more a prescriptive process, not a client- or patient-focused process, and making decisions, right?”* (Participant 13)*, “It was just a plan that was provided to me. There [were] no other, other options given”* (Participant 08), and *“I seldom ask questions. Because the more I ask questions, the more I get scared”* (Participant 14). Despite these observations, some Indigenous or racialized participants described receiving adequate information prior to enrolling in CDPs, and cited few concerns related to control or choice of fulfilling monitoring measures (e.g., *“I think [the CDP] is pretty good”* and *“[the CDP is] okay. I feel a little bit of trouble, but not too much”* (Participant 07). Similarly, for some, the benefits of the IMiD treatments outweighed any burdens associated with accessing them, “*because [lenalidomide] helps me. It helps me with my condition. You know?* (Participant 14).

Despite these experiences, most FCBP in this study categorized their participation in the CDP and compliance with the mandatory pregnancy monitoring and contraceptive mandates as a *“non-choice”*. Multiple participants described, *“giving away”* reproductive control or *“being forced to trade their rights”* in exchange for access to IMiD therapies. For instance, both Participants 06 and 11 shared:

*Yes, I could choose not to take this drug [and leave the program], but there's, but I mean, it's been proven as the most effective one on the market right now, to protect from the cancer coming back. So it's sort of a non-choice. You know? Yeah, I could not take it, but that's a non-choice.* (Participant 06)

Several participants highlighted that CDPs shift women’s control and power over their reproduction to others—nurses, laboratory technicians, physicians, and CDP staff, underlining that with this should come a sense of ‘duty’ and responsibility:

*The discomfort that the women are going through, while dealing with the fact that they're no longer in control of their own reproductive cycles. It's literally being given away, the control is being giving away to a medical company. And, we're saying, in exchange for our lives, we're going to give you control over our reproduction. So make sure that while you have that control, you treat it kindly and you treat it properly. If that makes sense (laughs).* (Participant 11)

Findings indicate that shifting responsibility and ‘control’ over women’s reproduction may unduly prioritize safeguarding against risks for fetal exposure, at the expense of women’s reproductive autonomy and agency. Further, participants’ perceptions of the requirements as “paternalistic” exemplify that, for them, CDPs see FCBPs as being untrustworthy, irresponsible, and/or incapable of managing their own reproduction. Against this backdrop, pharmaceutical regulatory and risk-minimization efforts appeared to be embedded in and influenced by broader historical, political, and social structures, which perpetuate women as incapable of making thoughtful and ‘responsible’ reproductive decisions.

#### Exacerbating the loss of reproductive identity and *“womanhood”*

Unexpected early menopause, physical and emotional side effects, and loss of fertility significantly affected many women in this study. For some, being able to have children represented a deeply engrained, valued, and vital component of their social and reproductive identities, sense of self or *“womanhood”* (Participant 13). One participant was diagnosed with MM while trying to get pregnant, and had to choose between urgent lifesaving treatments and pursuing fertility preservation, “*weighing, ‘do I need my kidney or do I want a baby in the future?’”* (Participant 07). For many, the prescriptive paternalism of CDPs compounded the sudden removal of their reproductive agency and physical ability to have children due to their MM diagnosis and treatment. Pregnancy monitoring requirements served as a (painful) monthly reminder of these complex and intertwined losses:

*There's a whole demeaning angle to it for moms, or women of childbearing age that are on [lenalidomide]. It's almost like putting salt in the wound. You know? Like, you can't have kids. You know you can't have kids. You shouldn't have kids. But we still make you go through this ridiculous survey, every single month and take pregnancy tests every month, as a reminder that you're taking a drug that would cause birth defects in children, but we know you can't have kids. It's nonsensical.* (Participant 04)

Women in this study were frequently caught in the middle – told by health care providers that they are infertile and menopausal, yet simultaneously required to prove they are not pregnant to the CDP in order to receive life-prolonging medication. These findings illustrate that participants may be enrolled in CDPs amidst grieving the unexpected and concurrent stripping of both their reproductive identity and agency, and that their participation in the hyper-vigilant monitoring practices of CDPs compounds this sense of loss, putting *“salt in the wound”* (Participant 04)*.*

### Systemic distrust of women in CDPs: *“Women can quite capably make informed decisions to avoid pregnancy”*

Pregnancy monitoring requirements and the mandated use of two forms of contraception were perceived by many study participants as symbolic of underlying distrust of women to effectively and autonomously manage their reproductive health. Although some participants described feeling *“comforted”* by the robust safety and pharmacovigilance measures of CDPs, almost all women in this research emphasized that these requirements questioned their capacity and competence to manage their reproduction, involved extensive work to ‘prove’ they were not pregnant, and underscored that FCBP cannot be trusted at their word.

#### Questioned capacity and competence: *“It makes me feel like a child”*

Almost all participants emphasized their understanding of the potential teratogenic risks and the importance of preventing pregnancy while taking IMiDs. Given this knowledge, women highlighted that imposed contraceptive and pregnancy monitoring requirements questioned their capacity and competence to effectively interpret risks, adhere to guidelines, and make ‘responsible’ reproductive decisions. The failure by CDPs and health authorities to concede that FCBP are also actively committed to and working towards preventing pregnancy was highlighted by one participant:

*I've had three kids. Like, I know what it's like to be pregnant. I know my own body. And I don't want to take these risks. Like, I'm not looking to do that. I want to prevent [pregnancy] as well. I'm on the same team…Yeah, I'm not, I’m not trying to work against them.* (Participant 11)

Similarly, other participants emphasized that educating participants on the potential risks and outlining guidelines/recommendations should be sufficient (e.g., *“I’ve got a handle on it. I understand the risk. I understand the consequence[s]”* (Participant 12). The hyper-vigilant approach to monitoring adopted within CDPs ultimately failed to acknowledge women as competent and capable of making rational, informed reproductive health decisions and/or respect their capacity to effectively recognize and mitigate risk. Experiences shared by multiple study participants illustrate this:

*In the past, those women didn't know that the drug was harmful to them. And they, and they were pregnant. Now, we know what the drug can do, and we can, you know, women can quite capably make informed decisions to avoid pregnancy.* (Participant 06)

*[The CDP] makes me feel like a child…it just feels like…I’m not trusted or something.* (Participant 15)

Several participants acknowledged the purpose of CDPs, as well as demonstrated a level of understanding of the legal, ethical, and historical reasons and contexts justifying the regulation of IMiD therapies. However, many opposed the intrusive and demeaning design and delivery of pregnancy monitoring and prevention practices. While some women in this study recognized these inherent constraints for the pharmaceutical industry and health authorities, they underlined prospects to adapt CDPs in a way that increasingly promote their agency and control:

*The pregnancy tests, every single month, that kind of, that felt condescending, like I wasn't smart enough to know (laugh), you know, how to take care of my own body and not get pregnant. I, I but I understand again, it's a legality thing, and they don't want their medication to harm a person, and their development and so, you kind of weigh the options and say, 'well, I get it.' (laugh) You know? 'While I'm on this, I'll, I’ll abide by your rules. I'll, I’ll take the pregnancy test’, but it was hard knowing that that was kind of being controlled for me.* (Participant 11)

#### The work and burden of ‘proof’

Our findings illustrate the substantial impact of CDPs on women’s reproductive health and wellbeing, as many women described accounts of extensive work to navigate the paternalistic nature of CDPs, and systemic lack of trust for FCBP to manage their reproductive health. Participants described at length the substantial mental, logistical, and emotional load and work involved in fulfilling pregnancy monitoring requirements (e.g., monthly surveys) and contraceptive measures for CDPs to ‘prove’ they were not pregnant. Despite completing these extensive exercises, many suggested that underlying gaps in trust remained. Many described these tasks as a burden, time-consuming, and *“a pain”*, substantially affecting their reproductive autonomy and psychosocial wellbeing. Notwithstanding the work involved in meeting requirements, participants felt that CDPs disregarded their current reproductive status (e.g., treatment-induced menopause), family planning desires/decisions (e.g., to not have children), and their longstanding (successful) reproductive health management, and fostered a sense of distrust. Failure to consider women’s reproductive agency, was indicated by one participant:

*Birth control always worked for me, so I kind of wonder why they wouldn't take that into consideration, when, as a woman, I know that I'm cautious, or that I'd know the effects that could happen. I'm cautious and I have it under control. So, I don't know why [they don’t take that into consideration].* (Participant 08)

Several participants described disclosing sensitive and personal information multiple times – to their oncologists, pharmacists and CDP representatives. Many were uncomfortable or embarrassed to reveal information about their sexual relationships and activities to CDP representatives, especially males, and described program staff as, *“robots, right? Reading the same questions over and over again”* (Participant 13). Further, the burden to record sexual activity and use multiple forms of contraception affected women’s sexual relationships and psychosocial wellbeing. For some, their enrolment in pregnancy prevention and monitoring initiatives made engaging in sexual activities feel too *“risky”* and *“tainted”*, significantly affecting their intimate experiences and relationships:

*I'm a grown woman of, like, currently, forty-eight years [old]. Like, I shouldn't be that scared of having sex with my husband (laugh).* (Participant 10)

In addition to overlooking women’s capacity to mitigate risk and control their reproduction without surveillance, many participants stressed that demands imposed on FCBP living with MM, their health care providers, pharmacists, as well as the broader health system, are disproportionate to the evidence on possible risks and a waste of finite health system resources. Several women described exceptions made by CDPs to use urine pregnancy tests at home during the COVID-19 pandemic and suggested that this option should be considered sufficient and sustained. Participants shared this recommendation as a feasible, private, and convenient strategy to ease the logistical and mental burden on FCBP. Moreover, it demonstrates a greater degree of trust in women, without requiring multiple physicians to confirm the participant is not pregnant.

### (Re)claiming control & agency: *“I can stop myself from having pregnancies, thank you very much”*

As previously outlined, FCBP described CDPs, particularly the routine pregnancy monitoring and prevention requirements, as *“paternalistic”, “intrusive”, “embarrassing”, “demeaning”,* and *“condescending”.* Despite the threats posed by CDPs to their reproductive agency and autonomy, many participants also indicated their commitment to social justice (*“I’m like, a trailblazer”* (Participant 13)) and their everyday strategies or moments of resistance and acts of advocacy for changes to CDP to (re)claim their reproductive agency and autonomy.

#### Subtle strategies of everyday resistance

Beyond efforts to volunteer, fundraise or seek/offer psychosocial support to others, many women in this study described resisting against the paternalistic requirements of CDPs. These moments of resistance ranged from subtle strategies to challenge CDP pregnancy prevention program requirements to more overt defiance or refusal to comply with measures. For instance, some participants described ignoring or “screening” phone calls from the CDP, because it served as a constant reminder of their terminal prognosis or expressing their discontent for the program through written and verbal communications with the CDP. Whereas others indicated occasions when they had “cheated” or did not recall using two forms of ‘acceptable’ methods of contraception, as per CDP requirements. Despite deriving some sense of control through small acts of resistance against the program, this also resulted in heightened stress for some women – women who were simultaneously managing significant psychosocial impacts imposed by their MM diagnosis and treatment:

*I'm like 'well then why do we need to use pregnancy control, if [my husband had] a vasectomy?' (laugh)…I got mad about this because it was just a dumb extra thing that I had to worry about. And then I'm doing surveys, and I'm like, 'I can't remember if I, if we used a condom every single time.'* (Participant 03)

*I'm not the kind of person to lie on a form, but honestly, like I would sit there, and I'd kind of have to lie about the, probably, in a year, five times we cheated and used a condom. But when I had those appointments [I was] really stressed about it.* (Participant 10)

Several participants also worked with their physician to dispute their status as FCBP. Although no participants in this study were successful, it further demonstrates their dedication and active efforts to oppose and challenge the paternalistic program criteria and monitoring guidelines.

#### Acts of advocacy for change

Multiple participants described engaging in volunteer work to raise awareness and funds for MM, as well as participating in and leading MM support and advocacy at both community and national levels. Several also emphasized the lack of available psychosocial services and supports and actively contributed to addressing this gap for other individuals, especially FCBP, living with and navigating MM. Moreover, many participants openly questioned the CDP requirements and advocated for changes, with a prominent focus on transforming the contraceptive requirements and approaches for pregnancy monitoring. Several women’s recommendations underscored the importance of ensuring that CDPs are more patient-centred, individualized, and reflective of the lived reproductive realities of FCBP. Many participants described their gratitude for IMiD treatments yet advocated that a universal approach to pregnancy monitoring is not an appropriate.

*I appreciate [lenalidomide], because it's keeping me alive, and keeping my myeloma at bay, before I need [the] next treatment. But, um I think the [controlled distribution] program needs to take a closer look at the population of younger, childbearing age[d] women, um and maybe determine things on a case-by-case basis, versus a blanket situation.* (Participant 04)

Multiple participants outlined practical recommendations and changes to CDPs that would help to foster an increased sense of control, comfort, and empowerment, while also adhering to safety standards. As previously highlighted, several FCBP proposed at-home pregnancy tests as a more convenient and private alternative to monthly blood serum tests. Others recommended having female CDP representatives to address their discomfort of sharing intimate relationship and sexual activity details. Further, increased opportunities for education to inform individuals of CDP eligibility criteria, particularly how long they would be considered a FCBP, and details on the CDP expectations prior to initiating treatment represented important recommendations outlined. In addition, some participants demanded that CDPs should evaluate their effectiveness to prevent pregnancy (particularly among women experiencing treatment-induced menopause), consistently strive to make programs less intrusive, and more critically and consciously consider the ethical and human rights implications of CDPs on FCBP. Participants acknowledged their concurrent commitment to advocating for change, alongside a resistance to shoulder this additional (and what they deemed unnecessary) burden:

*I ultimately want changes to [the controlled distribution program]. I don't know if that's going to happen, and I'm probably going to have to do more to make that happen. And that's frustrating too, is I don't want to have to be the one trying to push to make it happen.* (Participant 06)

Some participants also highlighted the prospects to and feasibility of introducing changes to CDPs, particularly increased and timely information, choice, and trust to support women to manage their own fertility and reproductive health. This reflects a shift from mandating the compliance of FCBP towards empowering and supporting women to make decisions. For many participants, their interest and motivation to participate in this qualitative study and to share their experiences of participating in a CDP were informed by and representative of their immense commitment to advocate for changes to CDPs and to improve opportunities for support for FCBP in the future.

*I noticed that, yeah, there was no mention of anyone, any women's input or feelings about any of this. It was just that 'Yeah, this program has stopped women having pregnancies.' And it's just like, I can stop myself from having pregnancies, thank you very much. I don't need you supervising me about that.* (Participant 06)

Overall, increased engagement of FCBP in the design and implementation of CDPs is paramount. Many women participating in this study emphasized their competency, capability, and willingness to autonomously manage their reproductive and health and that systemic change is needed to realize their reproductive autonomy and agency.

## Discussion

Our findings illustrate that controlled CDPs may restrict or undermine the reproductive agency and autonomy of FCBP living with MM due to CDP requirements that impinge on women’s capacity for ‘free’ choice and promote a culture of distrust in women’s competence and capacity to manage their reproductive health. Despite these structural challenges, our research underscores that women living with MM are not passive participants in their care, including their engagement in CDPs. Many demonstrated powerful acts of resistance and advocacy (including their participation in this research) and remained committed to social justice to (re)claim their reproductive autonomy and catalyze change for FCBP participating in CDPs in the future.

Our findings also suggest that CDPs are deeply entrenched in paternalistic motivations, prioritizing rigid risk-management efforts over women’s reproductive rights, autonomy, and mental health. For many women in this study, participation in CDPs represented a *“non-choice”* as they described compromises to their reproductive autonomy and agency to access lifesaving medications for MM. Moreover, some equated CDPs, especially pregnancy monitoring and prevention components, to the patriarchal policing of women’s bodies, sexuality, and the infringement of their reproductive rights (e.g., restrictive abortion laws and dress codes) [16, 29]. Our findings align with and extends research conducted by other scholars exploring ethical consequence for women participating in the *iPLEDGE* CDP, asserting that women’s reproductive rights are restricted solely based on their status as a FCBP and “*potential* to become pregnant and their *presumed* sexual activity” [47], p. 110 emphasis in original). Despite longstanding pregnancy prevention programs and the “proliferation of increasingly restrictive” risk management measures, fetal exposure to teratogens persists ([47], p. 110). Although some participants took comfort in the CDP safety measures, our findings also stress that pharmacovigilance efforts should not be at the expense of women’s reproductive autonomy, health, and wellbeing, especially given the limited evidence on their effectiveness to reduce fetal risk [15, 24, 49, 54]. For many participants, CDPs served as a “nagging” reminder of their terminal diagnosis, and the unexpected loss of their fertility and reproductive identity. Their adherence to pregnancy testing and monitoring, despite no longer being physically capable of having children, was summarized by a participant, as *“putting salt in the wound”*. Grounded in empirical qualitative data, our study offers novel insights on the lived reproductive injustices encountered by FCBP living with MM, a demographic that has largely been excluded in research to date on controlled distribution programs.

From a reproductive justice lens, interpretation of findings reinforce that the government and pharmaceutical industry inequitably weigh the rights of the unborn fetus versus those of FCBP living with MM. Further, it highlights that the design and delivery of CDPs should more adequately consider and accommodate participants’ lived reproductive realities. Many participants described the stress and work involved in adopting *two* forms of contraception, particularly amidst constraints/concerns associated with using oral contraceptives. Decisions prior to diagnosis to not have children and family planning, as well as treatment-induced menopause were flagged by participants as factors that should be considered in their eligibility and participation in pregnancy monitoring through CDPs. It is also critical to emphasize the pivotal role of other actors – drug manufacturers, national health authorities and regulators, pharmacies/pharmacists, and health care providers – in the design and implementation of CDPs and their risk minimization strategies. CDPs do not operate in isolation, but rather are one part of extensive regulatory and pharmacovigilance efforts [34]. Our findings offer important insights and connections between individual participants’ experiences of reproductive injustices to broader systems and structures that exert control over women’s bodies, deeply entrenched in and informed by historical, political, economic, social, and cultural contexts [16, 29, 32].

Critically, our findings demonstrate that CDPs are rooted in a systematic lack of trust and recognition of women’s capacity to make informed decisions and autonomously manage their fertility and reproductive health. The focus on women’s biological/physical health inadequately acknowledges how the extensive ‘burden of proof’ for FCBP affects their overall health and quality of life. Indeed, findings from our research reported elsewhere (Wigle, et al., unpublished), suggest that this approach to trolling women’s reproductive health has other far-reaching implications on their lived experiences given many of the practical/logistical constraints of participating in CDPs (e.g., limited freedom to travel and that family/social/work commitments revolve around completing monthly blood serum pregnant tests and accessing their medications). Qualitative research exploring the experiences of FCBP with isotretinoin risk reduction counselling also found that although participants fully understood the risk of teratogenic effects associated with the medication, counselling was deemed ‘anxiety-inducing’ and women were offered limited information on contraceptive options, particularly highly effective long-acting reversal contraception (e.g., contraceptive implant or intrauterine device) [58]. Innovative strategies to alleviate this work and burden on FCBP participating in CDPs is vital. For example, multiple participants recommended that adaptations offered during COVID-19 pandemic to allow for ‘at-home’ urine pregnancy tests be continued as a more private and convenient option to meet pregnancy monitoring requirements and reduce the burden on individuals and health systems. Recent literature on pregnancy monitoring for the *iPLEDGE* program also found that telemedicine visits and home pregnancy tests for participants on isotretinoin therapy increased access to therapy, avoided delays/disruptions, and encouraged “greater autonomy in individualizing testing for each patient” ([38], p. 2), underscoring this approach as an effective and feasible alternative to serum pregnancy tests. Given the feasibility and acceptability of alternatives to serum pregnancy test for FCBP, opportunities to sustain these adaptations should be considered.

Despite experiences of circumscribed reproductive agency and control, women in this study actively (re)claimed their autonomy through volunteering, providing psychosocial support to others living with MM, refusing to comply with what they felt were paternalistic mandates, participating in this research study to share their experiences and discontent, and advocating for changes to CDPs. Although participants had not (yet) realized tangible changes to CDPs or requirements, their actions reflect their perspectives on the social and reproductive injustices enacted by CDPs. In addition to fueling participants’ motivation to be engaged in this research and to recruit others, many study participants were passionate that findings be shared—following up frequently with the corresponding author (CP) to inquire about progress and the potential of this scholarship to enact systemic changes for women with MM. Many participants also advocated for their increased engagement in shaping CDPs, and establishing opportunities for psychosocial services and support, tailored to their specific experiences and needs. For instance, registries of FCBP enrolled in CDPs may encourage connections and social support given their overlapping experiences as FCBP, living with MM, and participating in a CDP.

This study offers insights connecting women’s individual-level reproductive injustices to broader, underlying systems of oppression. Given that reproductive justice was integrated as a framework for analysis and interpretation, rather than guiding study design and tools, opportunities to explicitly analyze other inequities experienced by women due to their social positions (e.g., race and class) were limited. Despite these constraints, we observed patterns that suggested some participants that self-identified as racialized and/or low-income were more likely to report limited engagement in broader healthcare decision-making, alongside their seeming “acceptance” of CDP requirements. While many participants perceived their enrolment in CDPs as a *“non-choice”*, Participants 07 and 14 (both racialized women) represented outliers, describing programs as *“good”* and that *“it wasn’t too much trouble”*. Substantial scholarship on systemic racism in the health system may offer context for interpretation of these results, as we recognize that “colonial, racist, and ableist structures” of health policy, academic research, health promotion, and patient care have contributed to lasting health and sexual and reproductive health inequities among racialized women [18, 21, 25]. For many Indigenous and racialized women, the historical and intergenerational trauma from structural racism, oppression, and medical experimentation have led to their exclusion from health care decisions, mistrust of health care providers/systems, and substantial reproductive injustices [18]. Our study findings suggest that future research on controlled distribution programs should move beyond consideration of age and gender, to specifically examine how individuals’ overlapping social identities, particularly race and class, shape women’s reproductive autonomy. Moreover, the integration of a reproductive justice lens to both research and health care delivery offers important connections between women’s reproductive health and social and structural determinants of health [25].

### Study limitations

There are several research study design and data generation considerations, which shaped our data analysis, interpretation, and synthesis of results. Firstly, we acknowledge that our sample of women living with multiple myeloma in this study have a relatively high socioeconomic status, including high levels of education and income. This may have shaped women’s knowledge of their sexual and reproductive health and rights, as well as limited our understanding of potential challenges posed by income status to participating in CDPs. Moreover, the perspectives of men receiving IMiD therapies are not represented in this qualitative study. Given IMiDs remain in sperm and may also contribute to fetal exposure to teratogens, men participating in controlled distribution programs are also required to adhere to specific criteria to access medications (e.g., wear a condom at every sexual encounter with a FCBP, and disclose medication use to partners of childbearing potential and associated risks) [11]. Examining men’s perspectives of participating in a CDP and its associated surveillance measures may offer a unique understanding of their experiences and offer important comparative context to the burden and systemic oppression experienced by FCBP. Moreover, these findings speak largely to the experiences of cisgender women and although gender diverse persons categorized as FCBP may experience similar issues related to their reproductive autonomy, they may also have distinct experiences and barriers (e.g., homophobia and transphobia), that shape their access to reproductive care and warrants further investigation [1, 5].

## Conclusions

Females of childbearing potential living with multiple myeloma represent a small proportion of the patient population, yet they experience unique challenges because of their multiple intersecting identities – including age, gender, and health status. Applying a reproductive justice theoretical framework illustrates that participation in existing CDPs undermines the reproductive autonomy and agency of FCBP, while compounding the loss of their fertility and reproductive identity. Although the importance of preventing pregnancy and minimizing fetal exposure to teratogens were widely recognized by all participants, pregnancy prevention and monitoring measures reflected a generalized lack of trust and respect for women’s competence and capacity to manage autonomously their reproductive health. Despite structural oppression and threats posed by CDPs, the women in our study described working hard to gain a sense of autonomy through resistance and advocacy to (re)claim their reproductive agency, and through their profound commitment to reproductive justice. Changes to the design and delivery of pregnancy monitoring and prevention requirements in existing CDPs appear achievable and should be considered to promote the reproductive autonomy and agency of women living with multiple myeloma.

## Supplementary Information

Supplementary Material 1.

## Data Availability

The data that support the findings of this study are not publicly available due to the ethics consent form indicating that participant data would not be shared outside of the research team. Data may contain information that could compromise the trust, confidentiality, and privacy of research participants, and thus will not be made publicly available.

## Abbreviations

CDP: Controlled distribution program
CMRG: Canadian Myeloma Research Group
FCBP: Females of childbearing potential
IMiD: Immunomodulatory agents
MM: Multiple myeloma
REMS: Risk Evaluation and Mitigation Strategy
SRH: Sexual and reproductive health

## Footnotes

1 Acceptable contraceptive methods are categorized as one ‘highly effective’ birth control methods (e.g., intrauterine device (IUD), hormonal methods (birth control, pills, hormonal patches, injections, vaginal rings, or implants), tubal ligation, partners’ vasectomy)), and an additional effective methods (e.g., male latex or synthetic condom, diaphragm, or cervical cap) [3], Bristol-Myers [10]. However, patient materials from the RevAid program specifically do not recommend hormonal methods for FCBP [40, 41], illustrating conflicting information for FCBP on contraceptive requirements.

2 In this paper, we use the terms “Females of Childbearing Potential” (FCBP) and “women” interchangeably. The term FCBP is employed by CDPs, and refers to females who are still menstruating, amenorrheic from previous treatments, or who are perimenopausal and is a key eligibility requirement for many controlled distribution programs. Exclusion criteria include menopause for more than 12 consecutive months, having a hysterectomy or bilateral oophorectomy, confirmed premature ovarian failure, XY genotype, Turner syndrome, uterine agenesis, and/or has not started to menstruate [40].

3 Reproductive identity is an emerging concept that examines how “individuals realize parenthood or non-parenthood, develop their reproductive identity, and integrate it into their overall sense of self” ([4], p. 245).

4 Abductive reasoning refers to a “form of creative inference, which involves the integration and justification of ideas to develop new knowledge” ([27], p. 2).

5 Category of income was defined according to Statistics Canada’s “low income cut-offs” [51].
