# Supplementary material for: Reproductive autonomy of women living with multiple myeloma participating in a pregnancy prevention program
Source: Reprod Health. 2025 Nov 20;22:235. doi: 10.1186/s12978-025-02182-z (PMC12632009; doi:10.1186/s12978-025-02182-z)
Supplement: Supplementary file 1 — Supplementary Material 1. [file 12978_2025_2182_MOESM1_ESM.docx]

**Supplementary File 1:** Summary of controlled distribution programs for multiple myeloma in North America

| **Controlled Distribution Programs** | **Program description** | **Medication** | **Eligibility of FCBP** | **Program Requirements for FCBP** |
| --- | --- | --- | --- | --- |
| **RevAid^TM^ (Canada)** | Controlled distribution program for IMiD medications, which outlined requirements for registered prescribers, patients, and pharmacists (RevAid, 2023a) | Lenalidomide, pomalidomide & thalidomide | Females who are still menstruating, amenorrheic from previous treatments, or who are perimenopausal are key eligibility requirements for the *RevAid* program. Exclusion criteria include natural menopause for more than 12 consecutive months, having a hysterectomy or bilateral oophorectomy, confirmed premature ovarian failure, XY genotype, Turner syndrome, uterine agenesis, and/or has not started to menstruate (RevAid, 2023a). | - **Pregnancy tests:** blood serum pregnancy tests (7-14 days and 24 hours before prescribed medication; every week during first 4 weeks of treatment; every 4 weeks for remainder course of treatment if regular/no menstrual periods or every 2 weeks if menstrual periods are irregular). - **Contraception:** use two effective methods of contraception every time have sex that could result in pregnancy, starting 4 weeks before initiating medication, while taking medication, during treatment interruptions and at least 4 weeks after stopping medications; or abstain from sexual contact that could result in pregnancy. Note, hormonal contraceptives are not recommended. - **Surveys:** complete monthly surveys immediately after pregnancy test by phone or computer. - **Additional safety rules:** Maximum 28 days per subscription, as compared to 84 days for males/non-FCBP, do not donate blood, and do not take medications if breastfeeding (RevAid, 2023a, 2023b). |
| **ApoSecure^TM^ (Canada)** | Controlled distribution program for Apo-Lenalidomide for registered prescribers, pharmacists, and patients that meet program conditions. | Apo-lenalidomide (generic alternative to *Revlimid*), Apo-pomalidomide (generic alternative to *Pomalyst*) | All females who are menstruating, amenorrheic from previous treatments, and/or perimenopausal (Apotex, 2020). | - **Contraception**: Mandatory use of two effective methods of contraception simultaneously (starting 4 weeks before treatment, during dose interruptions, during treatment, and for at least 4 weeks after discontinuing apo-lenalidomide treatment); or abstain from sexual contact that could result in pregnancy. - **Pregnancy testing**: Two initial negative pregnancy tests prior to initiating therapy (one at 7-14 days before therapy and one within 24 hours of prescribing drug); weekly pregnancy tests during first month of treatment and monthly thereafter (or every two weeks if menses are irregular), 4 weeks after discontinuation of treatment. Tests must be blood tests performed in licensed laboratories - FCBP that are currently pregnant or breastfeeding are not eligible for the ApoSecure program (Apotex, 2020). |
| **THALOMID Risk Evaluation and Mitigation Strategy (REMS) Program** | This program regulates the prescription, dispensing and use of thalidomide and requires registration by prescribers, pharmacies, and patients (Simin & Nagesh, 2020). It aims to prevent the risk of embryo-fetal exposure to *Thalomid* and inform key stakeholders on the serious risks and safe-use conditions of medications (Bristol-Myers Squibb, 2023b) | Thalidomide (*Thalomid*), Lenalidomide (*Revlimid*), Pomaldomide (*Pomalyst*) | Females are excluded if they are not pregnant and not able to get pregnant (e.g., natural menopause for 2+ years, removal of ovaries and/or uterus), or under the age of 18 and have not yet menstruated (with signature of parent/guardian) (Bristol-Myers Squibb, 2023a) | - **Contraception**: at least one highly effective method, and at least one additional effective method of birth control every time having sex that could result in pregnancy, starting at least 4 week before taking thalomid. - **Pregnancy tests:** pregnancy test from provider 10-14 days before receiving first prescription, again 24 hours before first prescription (Bristol-Myers Squibb, 2023a). - **Survey:** confidential survey form ensuring compliance with contraception, testing and drug therapy (Simin & Nagesh, 2020). |
